# AI-Assisted Design of Chemically Recyclable Polymers for Food Packaging

**DOI:** 10.3390/polym18060730

**Published:** 2026-03-17

**Authors:** Brandon K. Phan, Chiho Kim, Janhavi Nistane, Wei Xiong, Haoyu Chen, Woo Jin Jang, Farzad Gholami, Yongliang Su, Jerry Qi, Ryan Lively, Will Gutekunst, Rampi Ramprasad

**Affiliations:** 1 School of Materials Science and Engineering, Georgia Institute of Technology, Atlanta, GA 30332, USA; 2 School of Chemical and Biomolecular Engineering, Georgia Institute of Technology, Atlanta, GA 30332, USA

**Keywords:** polymer informatics, chemical recyclability, sustainable packaging

## Abstract

Polymer packaging plays a crucial role in food preservation but poses major challenges in recycling and environmental persistence. To address the need for sustainable, high-performance alternatives, we employed a polymer informatics workflow to identify single- and multi-layer drop-in replacements for polymer-based packaging materials. Machine learning (ML) models, trained on carefully curated polymer datasets, predicted eight key properties across a library of approximately 7.4 million ring-opening polymerization (ROP) polymers generated by virtual forward synthesis (VFS). Candidates were prioritized by the enthalpy of polymerization, a critical metric for chemical recyclability. This screening yielded thousands of promising candidates, demonstrating the feasibility of replacing diverse packaging architectures. We then experimentally validated poly(*p*-dioxanone) (poly-PDO), an existing ROP polymer whose barrier performance had not been previously reported. Validation showed that poly-PDO exhibits strong water barrier performance, mechanical and thermal properties consistent with predictions, and excellent chemical recyclability (∼95% monomer recovery), thereby meeting the design targets and underscoring its potential for sustainable packaging. These findings highlight the power of informatics-driven approaches to accelerate the discovery of sustainable polymers by uncovering opportunities in both existing and novel chemistries. Beyond identifying potential replacements, this work establishes a generalizable framework for navigating vast polymer design spaces under competing performance constraints. The results illustrate how data-driven polymer design can bridge the gap between sustainability concepts and experimentally realizable materials for real-world packaging applications.

## 1. Introduction

Food preservation is a contemporary challenge that requires materials providing both protection and sustainability. Appropriately optimized polymeric materials may serve as effective alternatives to conventional solutions. In particular, plastic packaging has fundamentally transformed global food supply chains by offering superior protective performance. These materials not only prolong product shelf life but also minimize food waste and support the worldwide distribution of perishable goods. Consequently, polymers have become widespread in modern industrial and consumer applications, forming the foundation of the packaging landscape [1,2,3,4,5,6,7,8].

**Figure 1:**
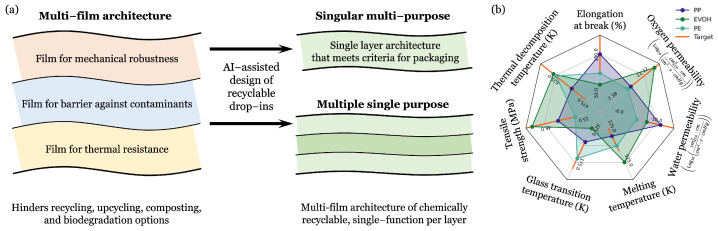
Sustainable food packaging design strategy and performance targets. (**a**) Conventional food packaging with complex multi-layer designs where each layer performs a specific function, complicating chemical recycling, can be replaced with two simplified, chemically recyclable alternatives, including a single-layer multi-purpose polymer and a multi-layer structure where each layer is a single-function, chemically recyclable polymer. (**b**) The design criteria are compared against the property profiles of common polymers (PP, EVOH, PE), highlighting the performance gaps that the newly designed polymer candidates must fill.

Despite these functional strengths, the very architecture and chemical persistence that make current packaging materials effective also represent major environmental challenges. Present-day packaging plastics often rely on a multi-layer architecture (Figure 1a), combining distinct, often non-compatible, layers for barrier, mechanical, and thermal functions [9,10,11]. While effective, these materials are notorious for their persistence in landfills, fragmenting into microplastics that contribute to long-term environmental pollution [12,13,14]. Furthermore, this multi-layer composition poses a significant obstacle to recycling since the chemically distinct layers must be separated, a time- and resource-intensive process [15,16]. The difficulty in finding suitable replacement materials that meet all necessary performance standards is highlighted by a comparison of the properties of currently used polymers, such as polypropylene (PP), ethylene vinyl alcohol copolymer (EVOH), and polyethylene (PE) (Figure 1b).

Recognizing these limitations, both academic and industrial efforts have increasingly focused on developing alternative packaging materials that enable sustainable end-of-life management while preserving the barrier, mechanical, and thermal performance required for food protection and processing applications [9,17]. Conventional multi-layer architectures, although highly effective in performance, are intrinsically difficult to recycle due to inseparable heterogeneous layers, motivating strategies that simplify material compositions or enable selective recovery of individual components [9,15]. A key thermodynamic parameter governing the feasibility of such chemical recycling is the enthalpy of polymerization, which determines the reversibility of polymerization–depolymerization equilibria [18]. Current sustainability approaches broadly include replacing persistent plastics with polymers that support closed-loop chemical recycling through reversible polymerization–depolymerization cycles, whose feasibility is governed in part by the enthalpy of polymerization, as well as materials designed for accelerated biodegradation under environmental or industrial composting conditions [19,20,21,22]. A more onerous design approach seeks to consolidate multi-film stacks into a single multi-functional layer, often referred to as a “drop-in replacement.” [23]. However, this strategy requires optimizing barrier performance, mechanical strength, and thermal robustness simultaneously, a complex design challenge that renewable polymers often struggle to meet [9,17,24].

To accelerate the discovery of these high-performance, sustainable alternatives, this work leverages recent advances in materials informatics methods to accelerate the design and property prediction of new polymers [25,26,27,28,29,30]. We developed a comprehensive polymer informatics workflow that incorporates computational modeling and machine learning (ML) tools to identify candidates that address the specific performance shortcomings of sustainable polymers. Through this workflow, we identified potential chemically recyclable polymers that may be created via ring-opening polymerization (ROP) [31] as candidate materials for simplified packaging architectures, including single-layer designs or recyclable multilayer systems [32,33]. This class of polymers with the ability to depolymerize and repolymerize not only reduces the need for new raw materials but also minimizes waste, thereby lessening the dependency on virgin resources and lowering the ecological footprint of polymer production. From this pool, we successfully synthesized and validated the key properties of poly(*p*-dioxanone) (poly-PDO), which shows promise as a recyclable barrier material for packaging applications with moderate oxygen barrier requirements [34,35,36].

## 2. Methods

### 2.1. AI-Assisted Design Workflow

**Figure 2:**
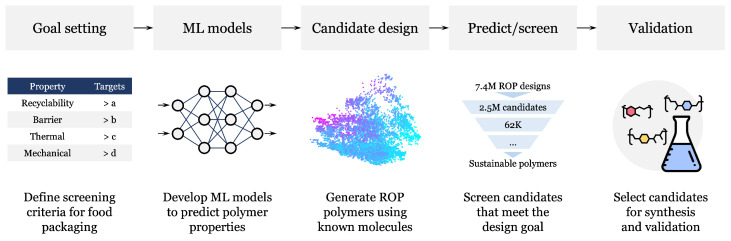
AI-assisted design workflow for recyclable polymer discovery. A schematic overview of the data-driven methodology illustrating an end-to-end materials informatics pipeline, progressing from goal setting and the definition of application-specific target properties to the curation of training datasets and the development of predictive ML models. The workflow includes candidate design via VFS, large-scale property prediction across the generated polymer space, and multi-stage screening to down-select candidates that meet recyclability, barrier performance, thermal properties, and mechanical constraints. The process concludes with experimental validation of promising polymer candidates.

Our overall strategy follows a materials informatics workflow designed to accelerate the discovery of sustainable polymers [18,37,38,39,40,41]. This approach, illustrated in Figure 2, systematically moves from defining target properties and curating necessary data to virtually synthesizing, predicting, and finally validating polymer candidates. All computational steps in this workflow, except for initial data collection and final experimental validation, were performed using PolymRize™ (version 0.27.0) [42], a standardized software for molecular and polymer informatics.

The workflow begins by defining the target performance criteria necessary for a single-layer, multi-functional packaging material, guided by the common packaging polymers shown in Figure 1b. These threshold values were selected to approximate the barrier performance ranges required for practical food packaging applications and were informed by permeability values reported for widely used packaging polymers such as PET and EVOH. These essential properties, including barrier, thermal, and mechanical requirements, are outlined in Table 1. Following the definition of these goals, the necessary data for the eight critical properties were compiled. The datasets used for model training consist primarily of experimental measurements collected from the literature, handbooks, and online databases. The only exception is the enthalpy of polymerization, for which additional values computed using density functional theory (DFT) were included alongside experimental measurements to expand the dataset coverage, as described in prior studies [43,44].

With the datasets in place, predictive ML models were developed for the eight target properties using a combination of Gaussian process regression (GPR) [45] and neural network (NN) architectures. GPR models were employed for training the enthalpy of polymerization model, while deep learning models were used for all other properties, following established protocols in prior studies [44,46]. Both single-task (ST) and multi-task (MT) training strategies were explored, where MT learning was used to jointly learn correlated properties to improve predictive accuracy. A summary of the dataset sizes, model types (GPR or NN), training strategies (ST or MT), and representative model performance metrics, reported in terms of root-mean-square error (RMSE) or order-of-magnitude error (OME), is provided in Table 2. Detailed descriptions of the dataset composition, feature representations, training configurations, and full model evaluation results, including parity plots, are provided in the Supplementary Information and in previously published works [43].

To efficiently explore the vast chemical space for sustainable solutions, we generated a large library of hypothetical, yet synthetically feasible [47], polymer designs. This virtual forward synthesis (VFS) process [18] was executed using the RxnChainer (Reaction Chainer) tool within the PolymRize™ platform [42]. Developing such extensive and chemically accurate datasets is a non-trivial process, requiring expertise in both reaction chemistry and informatics to encode precise rules. RxnChainer streamlines this complex workflow by systematically leveraging the established ROP reaction to combine about 30 million commercially available molecules. This targeted process successfully yielded a robust virtual library of approximately 7.4 million polymer candidates previously discussed [18] and released [48], which served as the comprehensive search space for the subsequent screening efforts in this work.

The ML models were then used to predict the eight target properties for the virtually synthesized polymer library, followed by screening against the criteria defined in Table 1. The selected enthalpy of polymerization range (−10 to −20 kJ/mol) reflects a thermodynamic window favorable for chemically recyclable polymers, where polymer formation remains sufficiently stable while still allowing efficient depolymerization under appropriate conditions [18]. Promising candidates were subsequently selected for experimental validation. The detailed synthesis and characterization procedures for the validated polymers are described in the Supplementary Information.

### 2.2. Synthesis of Poly-PDO from PDO

To experimentally validate a representative chemically recyclable polymer identified by the AI-assisted workflow as a promising candidate, poly-PDO was synthesized via ring-opening polymerization of PDO. Polymerization via ring-opening of PDO was conducted under bulk conditions with magnetic stirring in oven-dried 20 mL glass vials. PDO (5.0 g, 49 mmol) was first charged into a vial, which was then sealed with a rubber stopper. The initiator (benzyl alcohol) and catalyst (Sn(Oct)_2_ solution), each added in an amount of 10 μL (10 μmol, 2.0 × 10^−2^ mol % relative to the monomer), were subsequently introduced into the vial via syringe through the rubber stopper. The sealed vials were transferred to a silicone oil bath and immersed in the vial caps to ensure uniform thermal control. Polymerization was quenched at predetermined time intervals by removing the vials from the oil bath. A small aliquot was removed and dissolved in cooled CDCl_3_ to quench the reaction and allow ^1^H nuclear magnetic resonance analysis (^1^H NMR). The remaining material was dissolved in cooled CHCl_3_, followed by purification via precipitation in methanol. The resulting poly-PDO was collected and dried under reduced pressure. The average molecular weight of the final polymer was 26.6 kg/mol.

**Figure 3:**
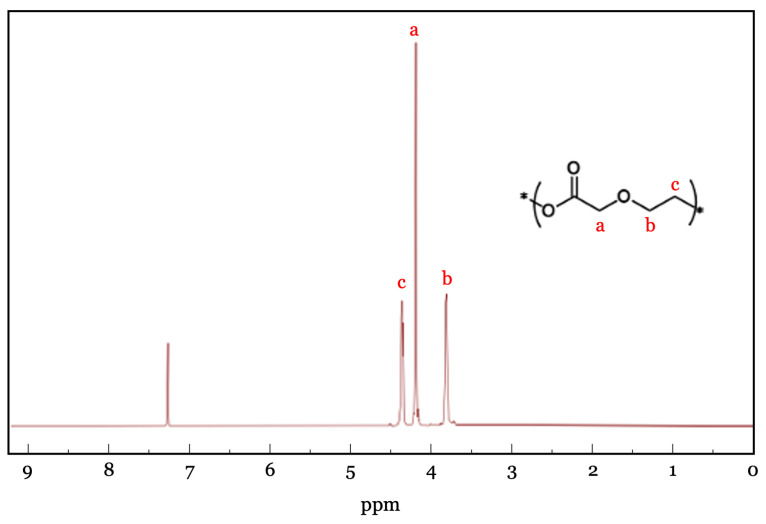
^1^H NMR spectra of poly-PDO in CDCl_3_.

The chemical structure and composition of the polymers were confirmed by ^1^H NMR spectroscopy in CHCl_3_ at room temperature (Figure 3). Spectra were acquired on Bruker Avance 400, 500, or 700 MHz instruments and referenced to the residual solvent signal of CHCl_3_ at δ 7.26 ppm. Characteristic resonances corresponding to the polymer backbone were clearly observed, confirming successful polymerization. Molecular weight distributions were further characterized by size-exclusion chromatography (SEC) using a Tosoh EcoSEC HLC-8320 GPC system (Tosoh Corporation, Tokyo, Japan) equipped with TSKgel SuperHZ-L columns. Measurements were performed using CHCl_3_ containing 0.25% NEt_3_ as eluent at a flow rate of 0.45 mL/min. Average molecular weights and dispersities were determined from refractive-index chromatograms and calibrated against PStQuick MP-M polystyrene standards.

## 3. Results and Discussion

### 3.1. Screening of ROP Polymer Candidates

Leveraging the predictive ML models, we rapidly predicted eight properties across the hypothetical ROP dataset comprising approximately 7.4 million candidates and systematically screened them against predefined criteria to identify promising candidate materials for simplified single-layer designs or recyclable multilayer systems. Among the eight target properties, the most critical initial filter was the enthalpy of polymerization, as it directly governs the feasibility of chemical recyclability through reversible depolymerization. Applying this criterion results in a substantial reduction of the search space. From the initial 7.4 million population, approximately 62,000 candidates fell within the targeted enthalpy of polymerization window of −10 to −20 kJ/mol while also exhibiting low prediction uncertainty below 15 kJ/mol. The uncertainty threshold was chosen to ensure that subsequent screening decisions were guided by predictions with sufficient confidence.

**Figure 4:**
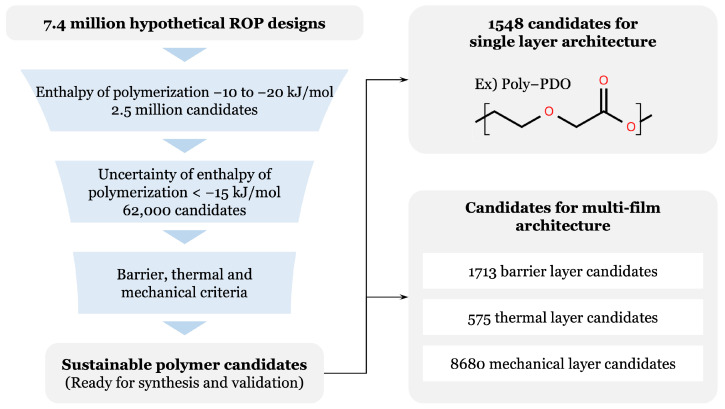
Schematics of the multi-stage screening workflow used to identify sustainable, chemically recyclable polymer candidates for both single-layer and multi-film packaging architectures. Starting from a virtual library of 7.4 million hypothetical ROP-derived polymer designs, candidates were first filtered by the target enthalpy of polymerization range, yielding 2.5 million polymers consistent with reversible polymerization thermodynamics. A second filter retained only candidates with low prediction uncertainty for the enthalpy of polymerization, reducing the pool to 62,000 polymers. The remaining candidates were then evaluated against the barrier, thermal, and mechanical criteria to produce a final set of sustainable polymers ready for synthesis and validation.

For this reduced subset, the remaining thermal, mechanical, and barrier properties were predicted using the trained ML models. This hierarchical screening strategy significantly reduced computational cost while preserving chemical diversity among the remaining candidates. The sequential funneling process, illustrated in Figure 4, demonstrates the feasibility of finding replacements for both single and multi-layer architectures. The predicted properties and structures for a selection of promising single-layer replacement candidates are presented in Table 3.

### 3.2. Experimental Validation of Film Performance

Based on a combination of favorable predicted properties and documented synthetic feasibility in prior literature, poly-PDO, which is shown as one of the representative example polymers in Figure 4, was selected as a potential recyclable barrier polymer for food packaging applications. Key properties of poly-PDO including enthalpy of polymerization, glass transition temperature, melting temperature, elongation at break, and tensile strength, have been previously reported in the literature, providing useful benchmarks for comparison between predicted and experimentally measured values. The availability of these reference data, together with the well-established synthetic accessibility of poly-PDO, motivated its selection as an initial validation system for the AI-assisted discovery workflow. While the virtual screening also identified additional candidates derived from less-documented monomers with promising predicted properties, poly-PDO was prioritized for experimental validation due to the availability of these literature benchmarks and its clear synthetic feasibility.

Poly-PDO was synthesized through the polymerization of *p*-dioxanone using Sn(Oct)_2_ as a catalyst. The resulting polymer structure was confirmed by ^1^H nuclear magnetic resonance (NMR) spectroscopy (Figure 3), and the number-average molecular weight ($M_{n}$) determined by SEC was 26.6 kg/mol with a dispersity of 1.54. While thermal and mechanical properties of poly-PDO have been reported previously, its gas and water vapor barrier properties, which are critical for evaluating suitability in food packaging contexts, have not been systematically investigated. Accordingly, the experimental validation in this work focused on addressing this key gap in the literature while also providing a comprehensive characterization of all relevant physical properties.

Thermal properties were rigorously evaluated using differential scanning calorimetry (DSC). The experimentally measured glass transition temperature of 257 K and melting temperature of 378 K were found to be in close agreement with the ML predictions of 261.9 K and 360.5 K, respectively, as well as with established literature ranges. Details of the instrumentation and measurement protocols are provided in the Supplementary Information. This agreement between predicted and measured thermal properties provides an initial and important validation of the predictive capability of the ML models for key thermophysical parameters. A comprehensive comparison of predicted values, experimental measurements, and available literature data for all eight target properties is summarized in Table 4, enabling a direct assessment of model performance and experimental fidelity. The thermal degradation temperature of poly-PDO, measured to be 487 K, providing a processing window of approximately 110 K above the experimental melting temperature. This temperature range is generally sufficient to enable melt processing operations such as extrusion or film formation under controlled conditions.

Poly-PDO demonstrated excellent barrier performance for water vapor, meeting the target goal. The ML prediction and experimental measurement aligned closely for water vapor permeability, classifying poly-PDO as a high-grade barrier that comfortably surpasses the set threshold. However, the oxygen barrier property remains a limitation, exhibiting one order higher permeability than the targeted design goal. Despite this constraint, poly-PDO is still well-suited for applications like fruit and salad packaging, where stringent oxygen barrier requirements are less critical [24].

The agreement between the predicted and experimentally measured mechanical properties (elongation at break and tensile strength) showed a discrepancy of approximately one order of magnitude (Table 4). While the predicted values met the design targets, the experimental results fell short of these expectations. This disparity may arise from several factors. Mechanical properties of polymers are known to exhibit substantial variability across different studies because they depend strongly not only on chemical structure but also on microstructure and processing history. Factors such as molecular weight and its distribution, crystallinity, residual catalyst content, sample preparation method, strain rate during testing, and environmental conditions can significantly influence the measured values. Consequently, literature data for the same polymer system often span a wide range of reported mechanical properties.

Such variability is also evident in previously reported measurements for poly-PDO [34,52], where a broad dispersion of mechanical property values has been observed across different studies. This dispersion suggests that experimental values may depend strongly on synthesis conditions, processing routes, and testing protocols. In addition, the training dataset used to develop the ML models in this work spans a very wide range of experimental values (0.005–289 MPa), covering several orders of magnitude. While the model captures general structure–property relationships across this broad property space, prediction uncertainty for specific systems is expected due to the heterogeneous nature of literature-derived datasets. Mechanical property prediction remains one of the challenging targets in polymer informatics because these properties are strongly influenced by microstructure and processing history, which are typically not encoded in structure-based molecular descriptors used in current ML models. The present results therefore highlight an important opportunity for future work. Incorporating descriptors that capture processing conditions, molecular weight characteristics, and microstructural features could further improve the predictive capability of ML models for mechanical properties.

### 3.3. Chemical Recyclability

**Figure 5:**
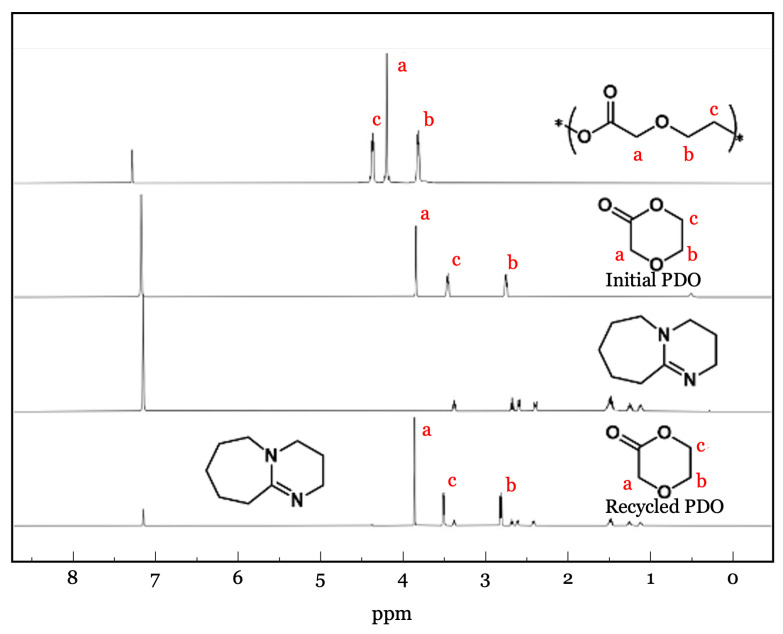
Overlaid ^1^H NMR spectra of pristine PDO, poly-PDO before depolymerization, and recycled PDO obtained after DBU-catalyzed depolymerization in benzene-*d*_6_ at 79 °C, demonstrating regeneration of the PDO monomer.

To experimentally confirm chemical recyclability, the depolymerization of poly-PDO was investigated using 1,8-diazabicycloundec-7-ene (DBU) as a catalyst in benzene solution at 79 °C. An oven-dried 5 mm thick sealed NMR tube was charged with poly-PDO (30 mg), DBU (10 mol% relative to repeating units), and a benzene-*d*_6_ (0.5 mL), sealed with a Teflon stopcock, and immersed in a silicone oil bath for up to 6 h. ^1^H NMR spectroscopy was used to monitor the depolymerization process by comparing the integrals of methylene resonances corresponding to the polymer and the regenerated monomer. This chemical depolymerization process exhibited high efficiency, achieving over 95% monomer recovery and approaching quantitative yields of ∼100% within 6 h, as confirmed by NMR analysis (Figure 5). The demonstrated near-complete recovery of the PDO monomer confirms that poly-PDO is a chemically recyclable polymer. When combined with its established biodegradability [52,53,54,55], poly-PDO offers multiple sustainable end-of-life pathways for food packaging applications, encompassing both closed-loop chemical recycling and biodegradation-based disposal strategies. Although benzene was used in this study as a convenient laboratory solvent to demonstrate the depolymerization concept, future work will explore greener solvent systems or solvent-free approaches to further improve the sustainability of the recycling process.

### 3.4. Practical Packaging Context and Structure–Property Considerations

From a packaging materials perspective, the performance of poly-PDO can be viewed within the broader landscape of commercial barrier polymers. Commodity packaging polymers such as PP generally exhibit relatively high oxygen permeability, while high-barrier materials such as EVOH provide significantly stronger oxygen barrier performance but often require multilayer structures and are difficult to recycle. The measured barrier properties of poly-PDO place it within an intermediate regime between these material classes, suggesting potential applicability in packaging scenarios requiring moderate oxygen barrier performance. In addition, the semicrystalline morphology of poly-PDO contributes to reduced gas permeability through the formation of crystalline domains that hinder diffusive transport, while the amorphous phase governs mechanical flexibility. This behavior can be rationalized by the regular polyester backbone of poly-PDO, which promotes semicrystalline packing that reduces free volume and slows gas diffusion, while the flexible ether linkage in the repeating unit contributes to chain mobility in the amorphous phase and supports mechanical ductility.

Hydrolytic degradation of poly(p-dioxanone) has been extensively studied in the biomedical polymer literature, where ester bond hydrolysis leads to gradual molecular weight reduction and mass loss in aqueous environments. Previous studies have shown that amorphous regions degrade preferentially while crystalline domains remain more stable during early stages of degradation [56]. The hydrolytic degradation behavior of poly-PDO has been investigated in aqueous environments, where molecular weight reduction and gradual mass loss occur due to ester bond hydrolysis [57]. These characteristics suggest that environmental conditions such as humidity and temperature may influence the long-term stability of poly-PDO films in packaging applications and therefore warrant further investigation in future studies.

The present results highlight a specific application space in which chemically recyclable polymers derived from ring-opening polymerization chemistries can provide promising combinations of barrier performance, thermal stability, and recyclability for packaging applications with moderate oxygen barrier requirements. The results further demonstrate the value of the AI-assisted discovery workflow in efficiently identifying experimentally testable candidates from extremely large polymer design spaces, thereby accelerating the exploration of sustainable packaging materials.

## 4. Conclusions

We successfully implemented a polymer informatics workflow that leverages predictive ML models and digital reactions to accelerate the discovery of sustainable packaging materials. This AI-assisted, data-driven strategy systematically screened an initial library of approximately 7.4 million ROP polymers, down-selecting to a focused set of promising single- and multi-layer polymer film architectures. The workflow was validated through the experimental synthesis and characterization of poly-PDO, a rediscovered ROP polymer. Its strong potential was confirmed by high-grade water barrier performance and excellent chemical recyclability, achieving over 95% monomer recovery within six hours. The close agreement between experimental measurements and ML predictions for poly-PDO supports the robustness of the predictive models. In contrast, discrepancies between predicted and measured mechanical properties highlight the need for further optimization of polymer samples and continued refinement of the ML models. Addressing these limitations will be essential to improve both experimental reliability and predictive accuracy in the design of practical polymers for sustainable applications. Although poly-PDO has been widely used in biomedical contexts due to its biocompatibility and hydrolytic degradability, additional regulatory evaluation and migration safety assessments would be required before its potential use in food-contact packaging applications.

More broadly, this study illustrates how informatics-driven materials discovery can uncover viable solutions that may be overlooked by conventional design paradigms. The identification of poly-PDO as a competitive packaging candidate emphasizes the value of re-evaluating known polymers through modern, data-centric frameworks. At the same time, the broader candidate pools identified in this work demonstrate the largely untapped potential of ROP-derived chemistries. The proposed workflow is not restricted to food packaging and can be readily adapted to other polymer-intensive sectors facing increasing sustainability constraints. By combining large-scale virtual screening with targeted experimental validation, this framework provides a scalable pathway for the rational design of polymers that balance performance, manufacturability, and end-of-life considerations. Future efforts will focus on improving dataset quality, enhancing mechanical property predictions, and incorporating processing constraints to further strengthen the practical impact of informatics-guided polymer design. Future work may also explore alternative recycling strategies for ROP-derived polymers, including melt-phase depolymerization or catalytic systems operating in greener solvent environments, to further enhance the sustainability of chemically recyclable polymer systems.

## Figures and Tables

**Table 1:** Screening criteria for sustainable packaging polymers.

Property	Desired Target
Enthalpy of polymerization	−10 to −20 kJ/mol
Water vapor permeability at 25 °C	<$10^{-9.3}$$\;{\mathrm{cm}}_{\mathrm{STP}}^{3}\cdot \mathrm{cm}/\left({\mathrm{cm}}^{2}\cdot s\cdot \mathrm{cmHg}\right)$ ^a^
Oxygen permeability at 25 °C	<$10^{-10.2}$$\;{\mathrm{cm}}_{\mathrm{STP}}^{3}\cdot \mathrm{cm}/\left({\mathrm{cm}}^{2}\cdot s\cdot \mathrm{cmHg}\right)$ ^a^
Glass transition temperature	<298 K
Melting temperature	>373 K
Degradation temperature	>473 K
Elongation at break	>$10^{2.17}$%
Tensile strength	>20 MPa

^a^ 1 ${\mathrm{cm}}_{\mathrm{STP}}^{3}\cdot \mathrm{cm}/\left({\mathrm{cm}}^{2}\cdot s\cdot \mathrm{cmHg}\right)$ = $10^{10}$ Barrer.

**Table 2:** Overview of training datasets, model architectures, and performance metrics. (ST: single-task learning, MT: multi-task learning, GPR: Gaussian process regression model, NN: neural network model, CV: cross-validation, RMSE: root-mean-square error, OME: order-of-magnitude error, a discrepancy where the predicted value differs from the reference value by a factor of ten or more).

Property	Datapoints	Model Type	Metric	CV Testset Error
Enthalpy of polymerization	109	MT-GPR	RMSE	0.067 kJ/mol
Water vapor permeability	36	MT-NN	OME	0.198
Oxygen permeability	747			
Glass transition temperature	8962	ST-NN	RMSE	31 K
Melting temperature	3938	ST-NN	RMSE	53 K
Degradation temperature	4563	ST-NN	RMSE	72 K
Elongation at break	1351	MT-NN	OME	0.37
Tensile strength	1023	MT-NN	RMSE	21 MPa

**Table 3:** Predicted property values for five selected candidate polymers (out of 1548) identified as promising materials for single-layer food packaging applications, and the structures of the selected candidates (bottom).

Polymer	C-1	C-2	C-3	C-4	C-5
Enthalpy of polymerization (kJ/mol)	−12.7	−17.08	−16.17	−16.93	−16.47
Water vapor permeability at 25 °C (${\mathrm{cm}}_{\mathrm{STP}}^{3}\cdot \mathrm{cm}/\left({\mathrm{cm}}^{2}\cdot s\cdot \mathrm{cmHg}\right)$)	$10^{-10.82}$	$10^{-9.72}$	$10^{-9.18}$	$10^{-9.33}$	$10^{-9.85}$
Oxygen permeability at 25 °C (${\mathrm{cm}}_{\mathrm{STP}}^{3}\cdot \mathrm{cm}/\left({\mathrm{cm}}^{2}\cdot s\cdot \mathrm{cmHg}\right)$)	$10^{-10.7}$	$10^{-10.51}$	$10^{-10.15}$	$10^{-10.04}$	$10^{-10.09}$
Glass transition temperature (K)	261.9	238.6	269.1	272.4	203.8
Melting temperature (K)	360.5	414.7	385.4	474.3	376.1
Degradation temperature (K)	524	479.8	538.6	605.8	507.4
Elongation at break (%)	191	151	204	162	174
Tensile strength (MPa)	32.62	33.54	55.5	24.83	35.13
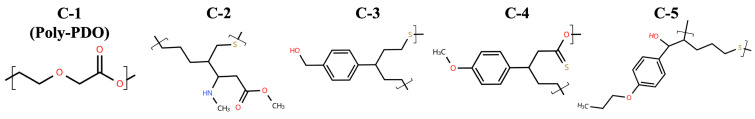					

**Table 4:** Comparison of poly-PDO predictions, measurements, and literature values. ^a^ [49], ^b^ [50], ^c^ [51], ^d^ [52], ^e^ [34].

Property	Prediction	Measured	
		This Work	Literature
Enthalpy of polymerization (kJ/mol)	−12.7±3.3	-	−13.8 ^a^
Water vapor permeability at 25 °C	$10^{-10.82\pm 0.2}$	$10^{-10.7}$	-
(${\mathrm{cm}}_{\mathrm{STP}}^{3}\cdot \mathrm{cm}/({\mathrm{cm}}^{2}\cdot s\cdot \mathrm{cmHg}$))			
Oxygen permeability at 25 °C	$10^{-10.7\pm 0.24}$	$10^{-9.0}$	-
(${\mathrm{cm}}_{\mathrm{STP}}^{3}\cdot \mathrm{cm}/({\mathrm{cm}}^{2}\cdot s\cdot \mathrm{cmHg}$))			
Glass transition temperature (K)	261.9±38	257	261 to 263 ^b,c^
Melting temperature (K)	360±29	378	363 to 397 ^b,c^
Degradation temperature (K)	524±55	487	-
Elongation at break (%)	$10^{2.28\pm 0.1}$	$10^{0.34}$	$10^{1}$ to 10^2.77 c,d^
Tensile strength (MPa)	32.6±6	3.3	3.9 to 60 ^d,e^

## Data Availability

The original contributions presented in this study are included in the article/Supplementary Material. Further inquiries can be directed to the corresponding author.

## Supplementary Materials

The following supporting information can be downloaded at: https://www.mdpi.com/article/10.3390/polym18060730/s1. The Supporting Information provides comprehensive details underlying the AI-assisted discovery and experimental validation presented in this work. It includes the definition of screening criteria for single-layer polymer replacements addressing barrier, thermal, and mechanical performance requirements, along with a summary of the datasets and machine learning model configurations used in the screening workflow. Additional analyses examine the effects of single-task and multi-task learning on water permeability prediction, including cross-validation strategies, data splitting protocols, and parity plots assessing model performance. Experimental validation data are also provided, including detailed gas transport measurement methodologies for oxygen and water vapor permeability, thermal characterization by differential scanning calorimetry, and mechanical testing following standardized tensile protocols. References [58,59] are cited in the supplementary materials.
