# Supplementary material for: AI-Assisted Design of Chemically Recyclable Polymers for Food Packaging
Source: Polymers (Basel). 2026 Mar 17;18(6):730. doi: 10.3390/polym18060730 (PMC13030112; doi:10.3390/polym18060730)
Supplement: Supplementary file 1 [file polymers-18-00730-s001.zip › polymers-4132906-supplementary.pdf]

Supplementary Information: AI-assisted design of chemically recyclable polymers for food packaging

Brandon K. Phan,† Chiho Kim,† Janhavi Nistane,† Wei Xiong,† Haoyu Chen,‡ Woo Jin Jang,‡ Farzad Gholami,† Yongliang Su,‡ Jerry Qi,† Ryan Lively,‡ Will Gutekunst,‡ and Rampi Ramprasad*,†

†*School of Materials Science and Engineering, Georgia Institute of Technology, Atlanta, Georgia 30332, United States*

‡*School of Chemical and Biomolecular Engineering, Georgia Institute of Technology, Atlanta, Georgia 30332, United States*

E-mail: rampi.ramprasad@mse.gatech.edu

Screening criteria

Table S1: Property requirements set for single, barrier, thermal, and mechanical layer replacements.

Property	Single layer target	Multi-film architecture targets		
		Barrier layer	Thermal layer	Mechanical layer
Enthalpy of polymerization (kJ/mol)	-10 to -20	-10 to -20	-10 to -20	-10 to -20
Water permeability $\left(\frac{cm^3_{STP}\cdot cm}{cm^2\cdot s\cdot cmHg}\right)$	$< 10^{-9.3}$	$< 10^{-9.3}$	$< 10^{-8.3}$	$< 10^{-8.3}$
Oxygen permeability $\left(\frac{cm^3_{STP}\cdot cm}{cm^2\cdot s\cdot cmHg}\right)$	$< 10^{-10.2}$	$< 10^{-10.2}$	$< 10^{-9.2}$	$< 10^{-9.2}$
Glass transition temperature (K)	< 298	< 323	< 200	< 323
Melting temperature (K)	> 373	> 373	> 373	> 373
Degradation temperature (K)	> 473	> 473	> 473	> 473
Elongation at break (Log_{10}(%))	> 2	> 2	> 2.17	> 2
Tensile strength (MPa)	> 20	> 10	> 10	> 30

Machine learning models

Table S2: Summary of datasets and model training setup.

Property class	Property	Datapoints	Data type	Model type	Total data
Thermodynamic	Enthalpy of polymerization (ΔH)	109	Expt.	MT-GPR	459
	Enthalpy of polymerization (ΔH)	350	Sim.		
Gas transport	$(\text{PDS})_x$ $x = [CH_4, CO_2, H_2, He, H_2O, O_2, N_2]$	5212	Expt.	MT-NN	7813
	$(\text{PDS})_y$ $y = [CH_4, CO_2, H_2O, O_2, N_2]$	2601	Sim.		
Thermal	Glass transition temperature (T_g)	8962	Expt.	ST-NN	8962
	Melting temperature (T_m)	3938	Expt.	ST-NN	3938
	Degradation temperature (T_d)	4563	Expt.	ST-NN	4563
Mechanical	Elongation at break (ε_B)	1351	Expt.	MT-NN	3809
	Tensile strength (σ_B)	1023	Expt.		
	Yieeld strength (σ_y)	407	Expt.		
	Young's Modulus (E)	1028	Expt.		

Our previous work assessed the effects of data fusion on the performative capabilities of permeability of common gases.[1] Here, we extend that body of work, following a similar analysis but with a particular focus on the prediction of water permeability. ST and MT models developed with PolymRize were used to predict the water permeability of 39 data points spanning 27 polymers, at various holdout train and test splits. These splits used

Table S3: Summary of model performance.

Property	Datapoints	Model type	Error metric	CV error
Enthalpy of polymerization (ΔH)	109	MT-GPR	RMSE	0.067 kJ/mol
Water vapor permeability (P_{H_2O})	36	MT-NN	OME	0.198
Oxygen permeability (P_{O_2})	747			
Glass transition temperature (T_g)	8962	ST-NN	RMSE	31 K
Melting temperature (T_m)	3938	ST-NN	RMSE	53 K
Degradation temperature (T_d)	4563	ST-NN	RMSE	72 K
Elongation at break (ε_B)	1351	MT-NN	OME	0.37
Tensile strength (σ_B)	1023	MT-NN	RMSE	21 MPa

stratified sampling based on the polymer SMILES string[2], giving a sense for how well the model forecasts unseen polymers. In addition, four random seed selections of the splits were used for the computation of the statistics of the model performance. The performance of the models was appraised using the coefficient of determination (R^2) and the order of magnitude error (OME).

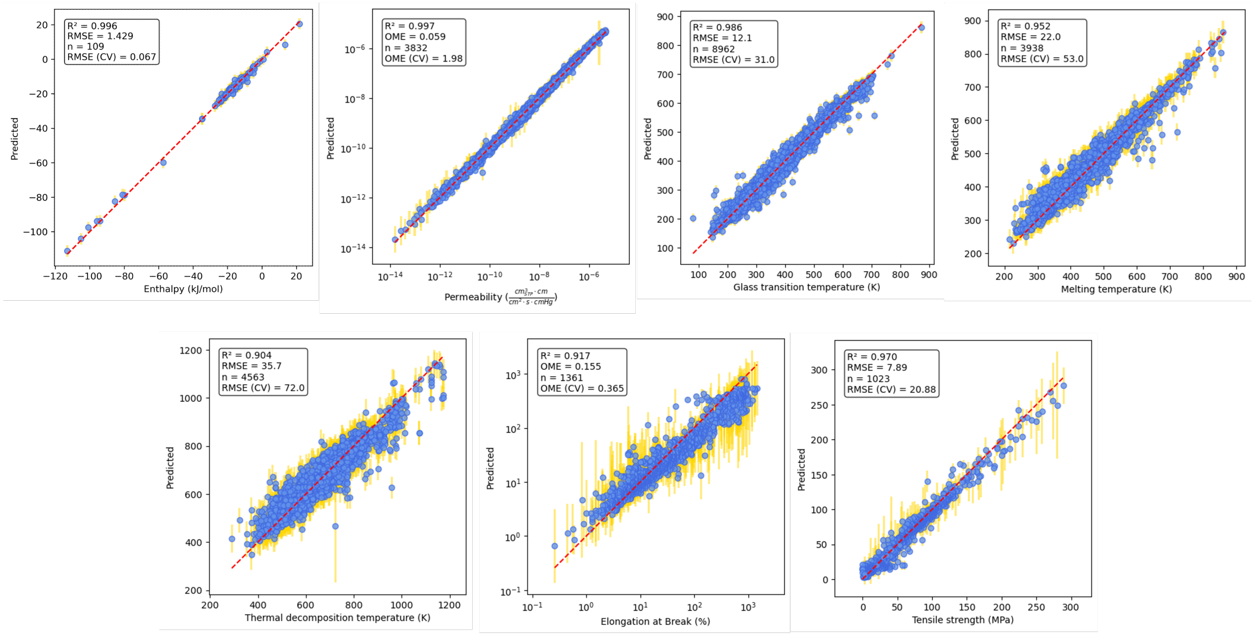

Figure S1: Parity plots of developed ML models. CV: Cross-validation

Experimental validation

Synthesis of poly-PDO from PDO

Ring-opening polymerization of PDO was performed in the bulk with magnetic stirring in oven-dried 20 mL vials. PDO (5.0 g, 49 mmol) was charged into a vial. The vial was sealed with a rubber stopper. The initiator (benzyl alcohol, 10 μL, 10 μmol, 2.0 $\times$ 10^{-2} mol % relative to monomer) and the catalyst ($Sn(Oct)_2$ solution, 10 μL, 10 μmol, 2.0 $\times$ 10^{-2} mol % relative to monomer), were subsequently injected into the vial through the rubber stopper with a syringe. The vials were then transferred to a silicone oil bath and immersed up to their caps. The vials were pulled from the reaction bath at predetermined intervals, and a small portion of each vial was separated and then dissolved in cooled $CDCl_3$ for reaction quenching and ^{1}H nuclear magnetic resonance (^{1}H NMR) measurement. The remaining portion of each vial was dissolved in cooled $CHCl_3$. The resulting PPDO was purified by precipitation from the $CHCl_3$ solution with methanol and dried in vacuo. The molecular weight of the resulting sample was 26.6 kg/mol.

The chemical structure and composition of the polymers were confirmed by ^{1}H NMR spectroscopy in $CHCl_3$ at room temperature. Spectra were acquired on Bruker Avance 400, 500, or 700 MHz instruments and referenced to the residual solvent signal of $CHCl_3$ at δ 7.26 ppm. Characteristic resonances for both comonomer units were clearly observed in the ^{1}H NMR spectra (Fig. S1). Size-exclusion chromatography (SEC) was performed on a Tosoh EcoSEC HLC-8320 GPC system equipped with TSKgel SuperHZ-L columns using $CHCl_3$ containing 0.25% NEt_3 as the eluent at a flow rate of 0.45 mL min^{-1}. Number-average molecular weights (M_n) and dispersities ($Đ$) were determined from refractive-index chromatograms and calibrated against PStQuick Mp-M polystyrene standards.

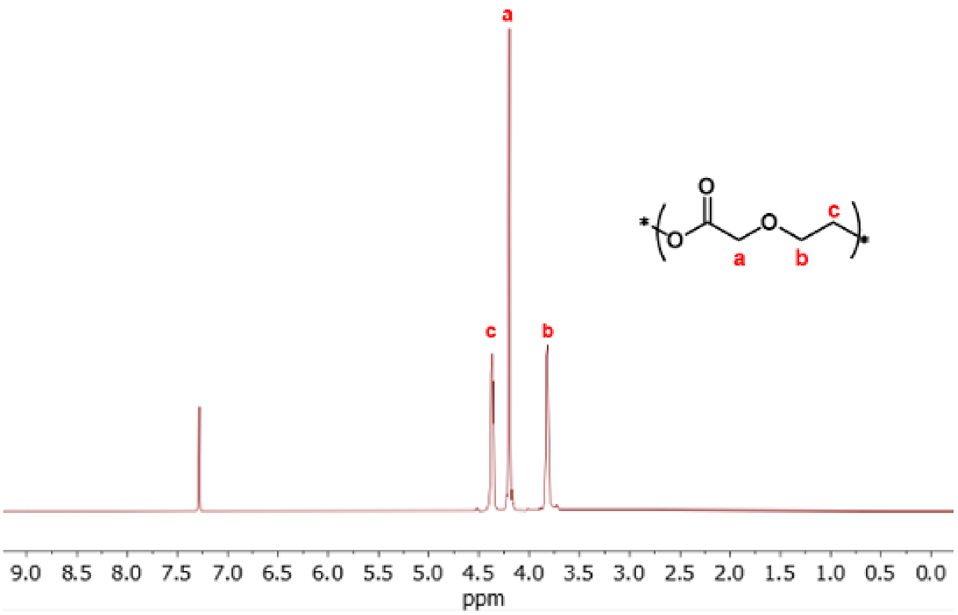

Figure S2: ^1H NMR spectra of poly-PDO in $CDCl_3$.

Depolymerization of poly-PDO

An oven-dried 5 mm thick wall NMR sealed sample was charged with poly-PDO (30 mg), DBU (10 mol% relative to repeating units), and Benzene-d6 (0.5 ml). The NMR tube was sealed by a Teflon stopcock and then immersed in a silicone oil bath at 79 °C for 6 hours before subjecting to ^1H NMR measurements. We integrate the methylene of the monomer and the polymer to obtain the integration, from which the degree of degradation was estimated.

Gas transport measurements

The poly-PDO membrane film for the gas transport measurements was prepared by first dissolving 1 wt% of the polymer in chloroform, and then filtered using 0.22 μm PTFE syringe filters (VWR), and then cooled to 277 K. A Matrimid support was fabricated following the procedure reported in the literature.[3] 0.5 mL of the dope solution was then spin-coated onto a cross-linked Matrimid support at 1,000 rpm for 2 minutes, resulting in an approximately 300 nm thin film of poly-PDO.

Oxygen permeation of the poly-PDO membrane was evaluated using a constant-pressure,

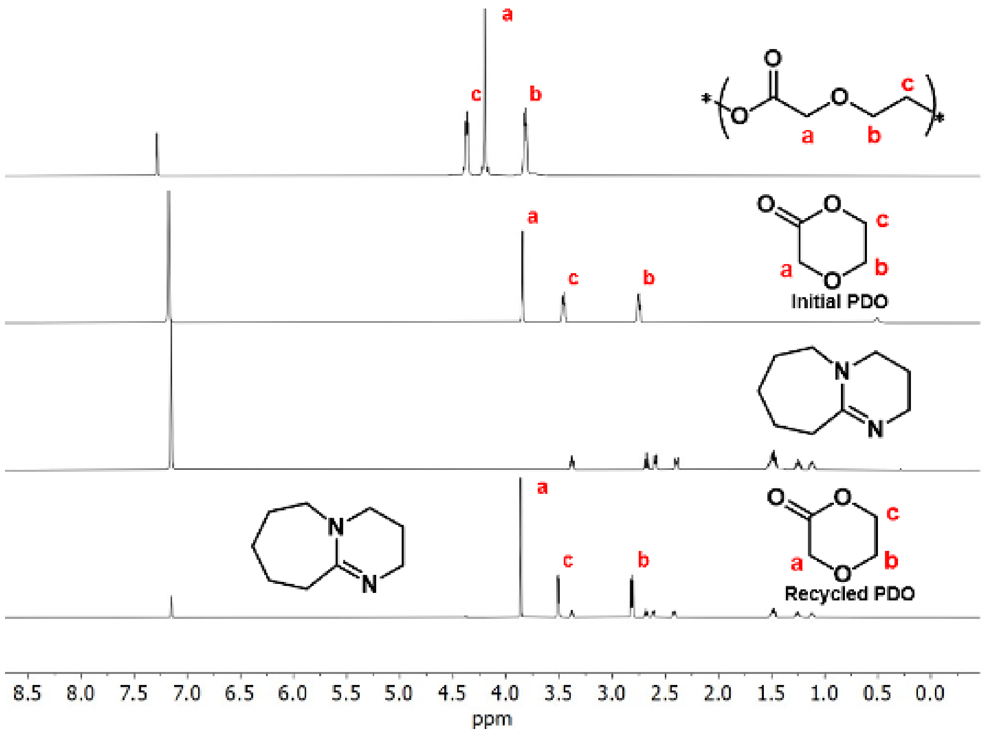

Figure S3: Overlays of ^{1}H NMR spectra of initial and recycled PDO and poly-PDO in benzene-d6.

single-gas permeation setup. A membrane sample with an effective area of 14.5 cm^2 was mounted in the sample cell, and both the upstream and downstream sides were evacuated with oxygen for 20 minutes. After evacuation, the upstream side and gas reservoir were isolated by closing the vent valve, and the reservoir was pressurized to 40 psi with pure oxygen at room temperature. The gas inlet valve was then opened, and the oxygen flow rate on the permeate side was determined by tracking the movement of a bubble through a 10 mL graduated pipette filled with Snoop solution. Each measurement was repeated five times, and the average value was used to calculate the oxygen permeability of the poly-PDO.

Water permeation experiments were conducted using a Wicke–Kallenbach cell with a poly-PDO (Fig. S4a). Helium (He) was used as the carrier gas and divided into three streams, each regulated by an individual mass flow controller (MFC). Two of the streams were combined to form the feed gas: one was passed through a water bubbler to generate humidified He, while the other supplied dry He. A three-way valve enabled mixing of the

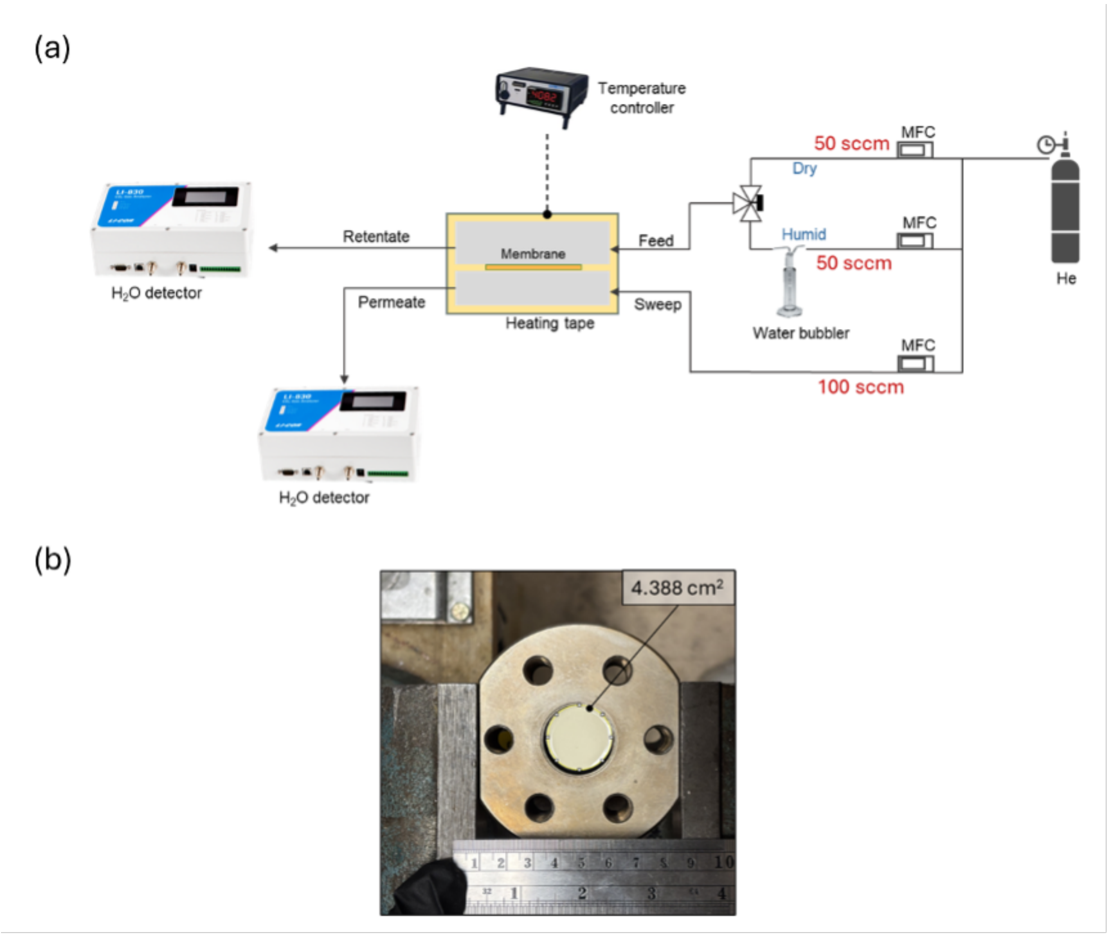

Figure S4: Gas permeability measurement setup. (a) Wicke–Kallenbach cell. (b) Top view of the membrane cell.

two streams, and the feed humidity was adjusted by varying the dry-to-humid flow ratio. The third stream provided dry He as the sweep gas. Both feed and sweep gases entered the membrane cell, where the PDO membrane was mounted. The retentate and permeate streams were directed to LI-850 gas analyzers (LI-COR Biosciences), which continuously measured CO_2 and H_2O reported concentrations. The membrane cell temperature was regulated by a thermocouple connected to a proportional–integral–derivative (PID) controller, ensuring stable operation at the target temperature. A top view of the membrane cell is shown in Fig. S4b. The effective membrane area was 4.388 cm^2, determined using ImageJ analysis of the mounted membrane image.

Thermal measurements

The glass transition temperature was measured using differential scanning calorimetry (DSC) under a nitrogen atmosphere with a heating rate of 10 °C/min, on a Mettler Toledo DSC 3+ (STARe).

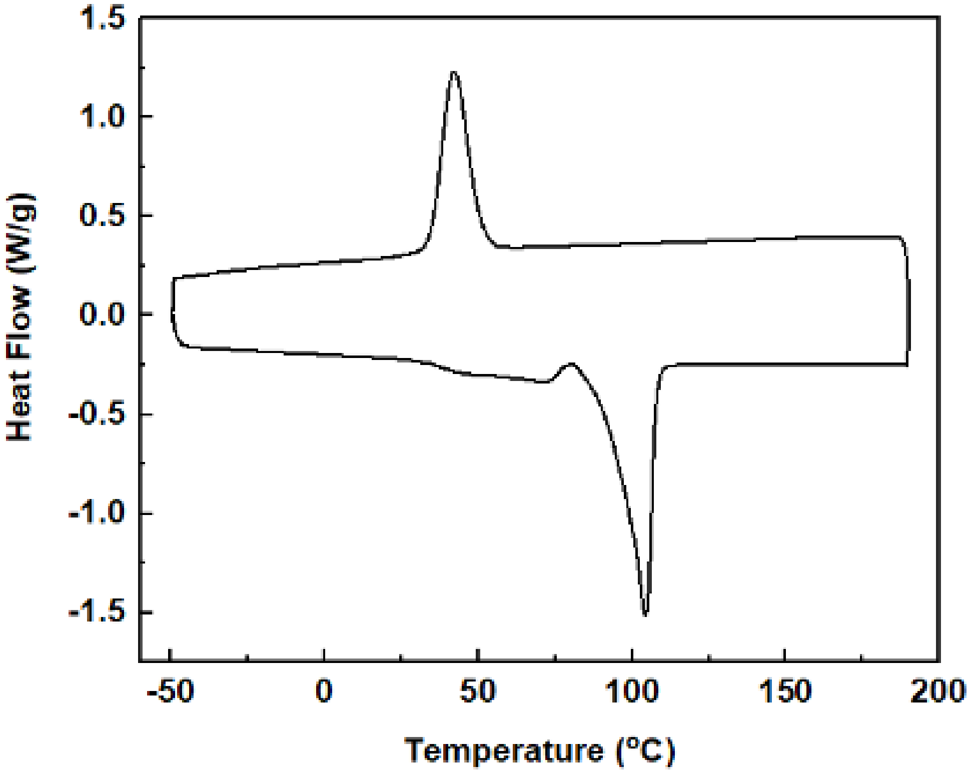

Figure S5: DSC curve of poly-PDO.

Mechanical measurements

The PDO films for mechanical measurements were formed via compression molding using a manual benchtop Carver press and sectioned into rectangular specimens following ASTM D882 guidelines. Before pressing, the material was preheated between the plates at 110 °C for 10 min to ensure uniform thermal equilibration. Pressure was then applied and maintained for an additional 15 min, facilitating complete melting and consolidation of the polymer. The resulting films possessed a nominal thickness of approximately 1 mm. Following molding,

the films were cooled under ambient laboratory conditions in air. Uniaxial tensile tests were conducted on a universal test machine (Insight 10, MTS Systems Corp., Eden Prairie, MN, USA) at a constant crosshead displacement rate of 5 $mm*min^{-}1$.

References

(1) Phan, B. K.; Shen, K.-H.; Gurnani, R.; Tran, H.; Lively, R.; Ramprasad, R. Gas permeability, diffusivity, and solubility in polymers: Simulation-experiment data fusion and multi-task machine learning. *npj Computational Materials* **2024**, *10*, 186.

(2) Weininger, D. SMILES, a chemical language and information system. 1. Introduction to methodology and encoding rules. *Journal of chemical information and computer sciences* **1988**, *28*, 31–36.

(3) Lee, Y. J.; Robinson, A. M.; Jang, W. J.; Ye, Z.; Ren, Y.; Xia, Y.; Lively, R. P. Prediction-Driven Selection of Microporous Polymer Membranes for Organic Solvent Reverse Osmosis. *Journal of Membrane Science* **2025**, 124475.
